# IUC24422-79 Patient satisfaction with penile prosthesis insertion following radical pelvic uro-oncological therapy

**DOI:** 10.1093/oncolo/oyaf248.049

**Published:** 2025-08-29

**Authors:** J Hay, S Michael, E Maher, A Haider, F Zarnani, V Modgil, I Pearce

**Affiliations:** Manchester Andrology Research Collaborative, Manchester Foundation Trust, United Kingdom; Manchester Andrology Research Collaborative, Manchester Foundation Trust, United Kingdom; Manchester Foundation Trust, United Kingdom; Manchester Foundation Trust, United Kingdom; Manchester Andrology Research Collaborative, Manchester Foundation Trust, United Kingdom; Manchester Andrology Research Collaborative, Manchester Foundation Trust, United Kingdom; Manchester Andrology Research Collaborative, Manchester Foundation Trust, United Kingdom

## Abstract

**Background:**

Radical treatment of pelvic uro-oncological pathology poses a significant challenge to sexual function, with recent survey data suggesting over 75% of men will have post-treatment erectile dysfunction (ED) and of these, almost 75% do not feel that their current treatment meets their needs in this area. In treatment-resistant ED, patients may be offered insertion of a penile prosthetic implant (PPI). We aimed to determine patient satisfaction with inflatable penile prosthesis insertion as a definitive treatment for post-oncological treatment erectile dysfunction.

**Methods:**

A cohort of 100 consecutive patients in a single Andrology unit who underwent penile surgery was anonymously surveyed to assess their post-operative satisfaction. Patients were asked to define their level of satisfaction in cosmetic appearance, sexual intercourse, ejaculation, and level of post-operative regret. From this cohort, we identified patients with PPI who had previously undergone radical uro-oncological therapy, surgical or otherwise.

**Results:**

The mean age of the patients was 62 years (61-70), shortest follow-up was 12 weeks. No patient expressed post-operative decision regret. 60% claimed they were “very happy” with their penile implant, with the remainder at least “happy.” On questioning whether these patients would undergo the same procedure, 80% stated “definitely yes,” with the remainder stating “possibly.” Interestingly, of concern was the degree of cosmesis, with over 50% of men being “unsure” or “unhappy” with the cosmetic appearance of their penis.

**Conclusions:**

It is well established that pelvic oncological resection or intervention carries a great risk to erectile function. It is promising from our data that PPIs can yield a positive benefit in these specific patients and restore a degree of sexual function where other conservative modalities have failed.

Penile cosmesis after insertion of PPI is complex and subjective, and managing expectations is clearly an important facet in the pre-operative period. PPI surgery offers a highly effective and acceptable modality for managing treatment-resistant ED in the selected, counselled patient post-intervention for pelvic malignancy. However, managing expectations on cosmesis as well as the degree of function remains an important element of the pre-operative encounter.

**Acknowledgments:**

Manchester Andrology Research Collaborative.

